# New Appliances and Things Medical

**Published:** 1894-10-27

**Authors:** 


### NEW APPLIANCES AND THINGS MEDICAL.

### ARMOUR AND CO.

(London Address: 59, Tooley Street, S.E.)

This firm send us a sample box of their various peptonising preparations, viz., granular pure soluble pepsin, granular pure powdered soluble pepsin, granular pure insoluble pepsin, pure pepsin tablets, glycerole of pepsin, essence of pepsin, pure pancreatin, glycerole of pancreatin, tablets of pancreatin and soda, peptonising food. Also the results of the peptonising process, viz., wine of beef peptones, and a sample of their famous extract of beef. We have experimented in the laboratory with these various peptonising materials and have found them invariably good and strong. For some time past we have been using Armour's peptonising tablets with soda for invalids' food with ever-increasing satisfaction. They are much liked by nurses, as they contain in themselves all that is needed. There is no measuring out, or weighing, but just the tablet broken up, added to the milk or other food and left to peptonise in the cold, or more quickly in a gentle heat. Another reason why we are in favour of Armour's preparation is that, as they prepare many hundreds of thousands of animals per annum for canning and other purposes, one is sure that the material for making pepsin is practically inexhaustible, and always fresh. There is no going about from one abattoir to another to collect the material, it is all there in one place, and the material can be used at once. Messrs. Armour and Co. are *fascile princeps* in their own line. Their preparations are undoubtedly the best in the market, and no better articles are to be obtained in the world.

